# Streptococcal Enzymes as Precision Tools Against Pathogenic IgG Autoantibodies in Small Vessel Vasculitis

**DOI:** 10.3389/fimmu.2019.02165

**Published:** 2019-09-20

**Authors:** Mårten Segelmark, Lars Björck

**Affiliations:** ^1^Nephrology, Department of Clinical Sciences, Lund University, Lund, Sweden; ^2^Infection Medicine, Department of Clinical Sciences, Lund University, Lund, Sweden

**Keywords:** vasculitis, ANCA, *Streptococcus pygenes*, anti-GBM antibody disease, autoantibodies

## Abstract

In primary systemic small vessel vasculitis autoantibodies are common and seem to play an important role in the pathogenesis. Autoantibodies in vasculitis are preferentially directed against components of the immune system or directly against components of the vessel wall. Plasmapheresis is often applied in emergency situationists when the function of vital organs is jeopardized, the level of clinical evidence to apply such therapy, however, varies between low and non-existing. Plasmapheresis is a blunt and unspecific instrument that requires several sessions to achieve a substantial reduction of autoantibody levels. IdeS and EndoS are two relatively recently discovered enzymes produced by *S. pyogenes*, that have a remarkable capacity to degrade and disarm IgG. They have shown positive results in several *in vivo* models of autoimmunity, and treatment with IdeS has successfully been used to inactivate HLA alloantibodies in patients undergoing renal transplantation. Both IdeS and EndoS have the potential to become precision tools to replace plasmapheresis in the treatment of vasculitic emergencies and a clinical trial of IdeS in anti-GBM vasculitis is now ongoing.

## Autoantibodies Are Common

The association of autoantibodies and inflammatory diseases was established more than 60 years ago (1); and now there are several hundred described specificities associated with different diseases. They are utilized for diagnostic purposes to differentiate between diseases and sometimes also as markers of disease activity; and they may participate in the pathogenesis (2). Elevated levels of disease associated autoantibodies can sometimes be found long before onset of symptoms and any diagnosis can be established (3). Autoantibodies can also be found in healthy humans (2, 4) and in mice raised under germfree conditions (5). Such natural autoantibodies are probably instrumental in removing cellular debris and seems to be protective of both arteriosclerosis and autoimmune disease. Most of these natural autoantibodies are thought to be of IgM class with limited avidity and specificity. However, it has been shown that healthy humans also have low levels of IgG antibodies (4) with highly restricted epitope specificity (6).

### Autoantibodies in Vasculitis

Vasculitides are broadly divided into primary and secondary forms, where primary vasculitides are diseases where inflamed blood vessel is the defining and most prominent feature. Primary vasculitides are further divided based on vessel size into large vessel, medium-sized vessel and small vessel vasculitis (7). Autoantibodies are common in all diseases in the small vessel group but are rare or at least not yet discovered in large and medium-sized vessel vasculitis (8, 9) (Table 1). Autoantibodies are also common in secondary forms of small vessel vasculitis, such as in systemic lupus erythematosus (1) and drug induced vasculits (10, 11), but in secondary vasculitis treatment should be aimed at underlying condition.

**Table 1:** Autoantibodies in small vessel vasculitis.

**Disease**	**Main autoantibody**	**Autoantigen**
Microscopic polyangiitis (MPA)	MPO-ANCA	Myeloperoxidase
Granulomatous polyangiitis (GPA, formerly Wegener's granulomatosis)	PR3-ANCA	Proteinase 3
Eosinophilic granulomatous polyangiitis (EGPA, formerly Churg-Strauss Syndrome)	MPO-ANCA	Myeloperoxidase
Anti-GBM disease (formerly Goodpasture's disease)	Anti-GBM	α3 chain of Type IV collagen
IgA-vasculitis (formerly Henoch-Schönlein purpura)	IgG/IgA anti-IgA	Degalactoslylated IgA
Cryoglobulinemic vasculitis	IgM anti-IgG	Polyclonal immunoglobulin G
Hypocomplementemic urticarial vasculitis	Anti-C1q	Complement factor C1q

### Autoantibodies Take Part in the Pathogenesis

The role of the autoantibodies in the pathogenesis, and the implicated pathogenic mechanisms, varies between the different diseases. In anti-GBM disease the binding of autoantibodies along the capillary wall of the glomeruli and alveoli start the complement cascade through the classical pathway and attract neutrophils through the C5a fragment (12, 13). Transfer experiments of eluted human antibodies injected into primates show that anti-GBM alone can mediate the disease (14), but there are also reports of anti-GBM models driven by T cells in agammaglobulinemic animals (15). Several *in vitro* studies indicate a role for anti-neutrophil cytoplasm antibodies (ANCA) in the pathogenesis of small vessel vasculitides such as microscopic polyangiitis (MPA) and granulomatosis with polyangiitis (GPA) (16, 17). There are many ANCA specificities in different autoimmune diseases but only myeloperoxidase (MPO) and proteinase 3 (PR3) that are expressed on the surface of primed neutrophils are major ANCA-antigens in vasculitis (8). The most compelling evidence for a role of ANCA in the pathogenesis comes from animal models of MPO-ANCA, where antibodies alone or antibody producing cells can transfer the disease (18). However, there are also data that do not support a direct role for ANCA in the pathogenesis; all purified IgG preparations from patients do not active neutrophils in a consistent manner (19, 20). IgA vasculitis (21) and cryoglobulinemic vasculitis (22) are immune complex mediated diseases, where polyclonal or monoclonal autoantibodies react with other immunoglobulins to form complexes. In urticarial vasculitis there are often autoantibodies directed to the complement factor C1q, which also lead to immune complex formation (23). Immune complexes activate complement primarily through the classical pathway which results in neutrophil influx and vessel wall damage (23). Physiochemical properties such as size and temperature determine where and when they will deposit, in urticarial vasculitis the direct targeting of the complement system also affect symptoms and signs.

## IdeS and EndoS

*Streptococcus pyogenes*, one of the most significant bacterial pathogens in humans, has evolved multiple mechanisms to avoid antibody attack and complement activation. IdeS, **I**mmunoglobulin G **d**egrading **e**nzyme of ***S****treptococcus pyogenes*, is a secreted cysteine proteinase which cleaves all four human IgG subclasses with a unique degree of specificity; apart from IgG no other substrate has been identified (24). Before cleavage can occur in the hinge region of the heavy chain to generate two Fc and one F (ab^1^)_2_ fragment the enzyme has to bind to the Fc region, and the remarkable specificity lies in this initial protein-protein interaction (25). *S. pyogenes* infects only humans, and from an evolutionary point of view it is noteworthy that the cleavage of IgG in other species is more restricted; in mice for instance subclasses 2a/c and 3 are sensitive, but not 1 and 2b (26).

Human IgG contains one N-linked glycan attached to Asn237 on the heavy chain (27). It is of great importance for effector functions such as complement activation and neutrophil recruitment. There are several bacterial enzymes that modifies N-linked glycans, but the first IgG specific glycan hydrolase to be described was EndoS which is also produced *Streptococcus pyogenes* (28). EndoS cleaves most of the carbohydrate moiety from IgG but leaves an N-acetylglucosamine with an alpha-linked fucose on protein backbone. EndoS treatment *in vitro* leads to reduced complement activation and phagocytosis of bacteria.

### IdeS and EndoS in Experimental Models

The species specificity hampers to use of IdeS in many rodent models. Not surprising is that pretreatment *in vitro* of pathogenic autoantibodies with IdeS can abolish disease in passive transfer models, such as immune thrombocytopenic purpura, neuromyelitis optica, and collagen induced arthritis (26, 29). What is more encouraging is that is that mice *in vivo* can be rescued from a lethal dose of rabbit anti-mouse thrombocytes and that arthritis induced by mouse IgG2a antibodies can be reduced in severity by IdeS *in vivo* (26). EndoS is easier to employ in experimental rodent models and have been shown to be effective to prevent or to treat disease in multiple settings, also in strains that spontaneously develop systemic inflammation (30).

The effect of IdeS and EndoS has also been investigated in experimental models of vasculitis. A mouse/rabbit model had been developed to mimic essential steps in the pathogenesis of anti-GBM disease. Here we took advantage of IgG species differences. Mice are first given a bolus dose of rabbit anti-mouse IgG; since rabbit IgG cannot activate mouse complement (31) this has no consequences. A week later, when there is no longer any circulating rabbit IgG, the animals are challenged with mouse-anti rabbit IgG. This leads to a dose-dependent renal injury mediated by complement induced neutrophil recruitment. When IdeS was given between the two IgG injections, it completely inhibited the development of proteinuria. Histological examinations confirmed that Fc fragments but not F (ab')2 fragment had been removed from the GBM. This was accompanied by a reduction in the deposition of complement and influx of neutrophils in the glomeruli. EndoS was also employed in this model, even though the setup is not ideal for testing EndoS a positive effect was seen.

EndoS has also been used in a model of ANCA associated vasculitis (32). Pre-treatment of human MPO-ANCA containing IgG with EndoS prevented neutrophil respiratory burst. When mouse anti-MPO was exposed to EndoS before injection into mice, the antibodies did not induce disease. In addition, when EndoS was given to the mice after challenge with anti-MPO IgG antibodies this attenuated the disease (32).

### IdeS in Humans

In a phase I trial, IdeS was given in different doses to healthy human volunteers (33). Doses as low as 0.12 mg/kg body weight led to a complete cleavage of not only plasma IgG but the entire extracellular IgG pool in all subjects without any obvious side effects. Intact IgG started to reappear after a few days and reached pre-treatment levels within a month. Varying titer of anti-IdeS antibodies were detected in the healthy volunteers; these levels rose after IdeS infusion, peaked after 2 weeks and was back to pre-treatment levels after 6 months.

IdeS is being developed as a pharmaceutical agent by Hansa-Biopharma; the non-proprietary name for the compound is Imlifidase. It has been tested in clinical trials to enable transplantation in patients with multiple HLA alloantibodies (34). A single dose given prior to transplantation enabled transplantations in 24 out 25 such sensitized patients who if they ultimately receive a kidney at all, may have to wait for years for a matching kidney (35). In many of the patients HLA antibodies rebounded, but 6 months after transplantation all 24 patients had functioning grafts.

IdeS like all other therapies can have side effects, total depletion of IgG take away an important protection against microorganisms. This is done also by plasma exchange but not as effective, on the other hand IdeS treated patients still have intact levels of IgA and IgM. Most individuals have measurable levels of anti-IdeS this introduces the risk of immune-complex formation and the development serum sickness. Furthermore, there is a theoretical risk of formation of neo-eptiopes by IdeS cleavage which can trigger autoantibody formation to IgG bound to different surfaces. Vigilance is always needed when introducing new pharma.

### The Potential Role IdeS and EndoS in Treatment of Human Vasculitis

The rapid depletion of autoantibodies that can be achieved by IdeS is potentially beneficial in acute settings where vasculits threatens the function of vital organs. Today plasma exchange or immunadsorption therapy is used to lower levels of pathogenic IgG antibodies in such settings (36). With plasma exchange only about one third of the total body IgG is removed in each session. That means that it takes several days to reduce the levels with one order of magnitude, and many times a greater reduction is needed. Immunadsorption is more effective, but so far there are no randomized trials showing that this therapy leads to an improved clinical outcome in any condition, as compared to standard plasma exchange. The question is whether depleting autoantibodies with IdeS or disarming them with EndoS would make a clinical meaningful difference.

Most patients with anti-GBM disease have rapidly progressive glomerulonephritis (13). The standard therapy today is the combination of cyclophosphamide to stop autoantibody production, plasma exchange to remove autoantibodies and steroids to dampen inflammation (37, 38). This therapy is effective if started early, but most patients are diagnosed late. More than 2/3 are diagnosed when glomerular filtration rate is below 15 ml/min and in such cases <10% achieve renal survival. Anti-GBM disease is in most cases a monophasic disease, where autoantibodies are only produced during a few months. This period is substantially shortened by immunosuppression (39). We treated three patients on compassionate basis with refractory anti-GBM disease after an individual permit from the Swedish Medicinal Agency (40). All there were dialysis dependent and had high levels of circulating anti-GBM despite intense plasma-exchange. In all three cases anti-GBM levels dropped to levels within the normal range. Using Fc-gamma specific antisera we could show that IdeS had cleaved IgG bound to the kidney. None of three, however, regained kidney function enough to stop dialysis. We are now conducting an investigator driven clinical trial (EdraCT2016-004082-39) where 0.25 mg/kg of imlifidase (IdeS) is given as a single injection early in the course. Anti-GBM disease is rare, and considering inclusion and exclusion criteria, we need a very large catchment area to include the goal of 15 patients. So far 15 tertiary referral hospitals in major European cities participate in the study and 11 out 15 patients have been included.

In ANCA associated vasculitis plasma exchange is often used when complicated by rapidly progressive glomerulonephritis with severe renal failure or alveolar hemorrhage with respiratory distress (41). However, the use of plasma exchange in ANCA vasculitis is controversial. The MEPEX study published in 2003, show positive effect on 1-year renal survival (42). The more recently conducted PEXIVAS trial, that is so far only available as congress abstracts, did not show any benefit of plasma exchange, neither in cases with rapidly progressive glomerulonephritis nor in cases with alveolar hemorrhage. The reason for the negative results could be that plasma exchange is not effective enough to lower autoantibody levels in acute settings. In such cases treatment with IdeS or EndoS would be attractive treatment options. On the other hand, it also possible that the autoantibodies only play a minor role in the pathogenesis and that their removal does not lead to any meaningful clinical effect.

IgA vasculitis, urticarial vasculitis and cryoglobulinemic vasculitis are all considered to be immune complex mediated diseases. Plasma exchange is being used in all three diseases, but the evidence level is low (36, 43, 44). The potential effect of IdeS and EndoS is only speculative and might not even be beneficial. IgA vasculitis is the most common form of primary systemic vasculitis in children. The yearly incidence is reported to be as high as 176 per million children (45). Most cases are, however, mild and heal without any treatment. The disease is much less common in adults, but on the other hand also more severe. The high rate of spontaneous recovery makes clinical trials difficult. IgA is not effective when it comes to complement activation; the presence of IgG in the complexes may therefore be instrumental for the development of vasculitis.

In cryoglobulinemic vasculitis IgG is the target of the autoantibodies. Removing them could also alleviate the inflammation. However, the short duration of the effect would only provide transient benefit, and severe vasculitic crises are rare in cryoglobulinemic vasculitis. The same is true for urticarial vasculitis. The role of the autoantibodies against complement component C1q is uncertain, and it must be kept in mind that F (ab')2 fragments can continue to form immune complexes also after losing their Fc tail.

## Conclusion

IgG class autoantibodies are common in primary small vessel vasculitis and they seem to participate in the pathogenesis. Today plasma exchange is often employed to reduce levels, but this treatment is unselective and rather ineffective. IdeS and EndoS are novel precision tools the rapidly either cleaves or disarms the IgG molecules. Whether this would lead to meaningful clinical responses remains to be determined in each individual disease. Anti-GBM disease, where the pathogenesis seems to be most straight forward, is first in line and a clinical trial is already ongoing.

## Author Contributions

MS wrote the first draft of the manuscript and then both authors contributed on an equal basis.

### Conflict of Interest

Hansa Biopharma AB owns the commercial rights for IdeS and EndoS as pharmaceuticals, and LB is listed as an inventor on the patents. MS and LB received research grants from Hansa Biopharma AB, and LB has an extensive research contract with the company.
